# Citizen science expanding knowledge: a new record of the lizard *Heterodactylusimbricatus* (Squamata, Gymnophthalmidae) in south-eastern Brazil

**DOI:** 10.3897/BDJ.11.e107929

**Published:** 2023-11-23

**Authors:** Cássio Zocca, André Felipe Barreto-Lima, Dulce Barbosa Daleprane, Natalia P. Ghilardi-Lopes

**Affiliations:** 1 Projeto Bromélias, Postgraduate Program in Animal Biology, Federal University of Espírito Santo, Vitória, Brazil Projeto Bromélias, Postgraduate Program in Animal Biology, Federal University of Espírito Santo Vitória Brazil; 2 National Institute of the Atlantic Forest, Santa Teresa, Brazil National Institute of the Atlantic Forest Santa Teresa Brazil; 3 Federal University of ABC, São Bernardo do Campo, Brazil Federal University of ABC São Bernardo do Campo Brazil

**Keywords:** community engagement, conservation, geographic distribution, local communities, volunteer citizens

## Abstract

**Background:**

Through citizen science projects, like Projeto Bromélias, community members contribute valuable data on species diversity, notably those with low detectability like the *Heterodactylusimbricatus* lizard. A recent observation in the State of Espírito Santo (south-eastern Brazil), amidst coffee and eucalyptus crops, highlights the utility of widespread technology use in tracking and documenting wildlife. Such initiatives are especially beneficial for mapping the distribution of rare, endemic or endangered reptiles. Therefore, we advocate for more citizen science initiatives near protected areas, involving local communities.

**New information:**

We provide a new record for the species *Heterodactylusimbricatus*, a microteiid lizard of low detectability from the Atlantic Forest of south-eastern Brazil. *Heterodactylusimbricatus* (Rio de Janeiro Teiid) was recorded near the protected area "Reserva Biológica Augusto Ruschi" by a citizen volunteer who contributes herpetofauna records to the Bromelias Project (https://www.inaturalist.org/projects/projeto-bromelias). *Heterodactylusimbricatus* is a very poorly-known species in the localities where it occurs, probably due to its fossorial habit, genera's restricted occurrence range, habitat specificity and the absence of proper survey methods fitted to fossorial species, such as the utilisation of pitfall traps. By publishing the records of volunteer citizens, we hope that more people will contribute to increase the knowledge of biodiversity in the mountainous region of Espírito Santo State and expand our collective knowledge.

## Introduction

Citizen science projects can provide valuable scientific data through community participation in scientific research (Bonney et al. 2009a, Bonney et al. 2009b, Miller-Rushing et al. 2012, Burgess et al. 2017, Niemiller et al. 2021), which is essential for conservation (Cooper et al. 2007, Theobald et al. 2015, Parker et al. 2018, Aristeidou et al. 2021) and natural resource management (Charnley et al. 2008). For example, through citizen science, it is possible to assess species diversity and distribution (Elith and Leathwick 2009, Gómez-Hoyos et al. 2018,Phillips 2016), and evaluate their biological responses to environmental change (Lepetz et al. 2009).

Such an approach can be advantageous for gathering occurrence data of species with low detectability, such as lizards of the genus *Heterodactylus* (Rodrigues et al. 2007, Rodrigues et al. 2009a). This genus includes three species of limb-reduced fossorial lizards with very elongate bodies and tails, whose range is restricted to Brazil, in the States of Bahia (*Heterodactylusseptentrionalis* Rodrigues, Freitas and Silva, 2009), Minas Gerais, Rio de Janeiro, São Paulo, and Espírito Santo (*Heterodactylusimbricatus* Spix, 1825 and *Heterodactyluslundii* Reinhardt and Lutken, 1862). As these species rely on protected areas, local changes in the environment, even at small scales, can lead to their local extinctions (Román-Cuesta and Martínez-Vilalta 2006).

*Heterodactylusimbricatus* is a rare cryptozoic lizard with low detectability due to its fossorial habit, spending most of the time under the leaf litter. This species occur at high altitudes in the Atlantic Rainforest and gallery forests in the Brazilian Cerrado (Von Hering 1898, Rocha et al. 2004, Sendas and Araújo 2004, Dixo and Verdade 2006, Dixo and Metzger 2009, Rodrigues et al. 2007, Rodrigues et al. 2009a, Rodrigues et al. 2009b, Marques et al. 2009, Novelli et al. 2011, Oliveira et al. 2020). In the present article, we report a new occurrence record of the lizard *H.imbricatus* in the State of Espírito Santo, identified by a participant of Projeto Bromélias citizen science project.

The Projeto Bromélias (Bromelias Project - https://www.inaturalist.org/projects/projeto-bromelias) has been developing community engagement in science around the protected area "Reserva Biológica Augusto Ruschi", in the Municipality of Santa Teresa, State of Espírito Santo, since 2012. This is done through visits to residents, distribution of educational materials (brochures, cards and stickers), itinerant events, and photo exhibitions that highlight the importance of wilderness, raise awareness and encourage people to change their attitudes towards nature. In addition, the dissemination of the project and popularisation of science was also carried out through social media on the internet, to engage the community to register, through geolocated photographs, the amphibian species in their surroundings, using smartphone devices.

## Materials and methods

**Figure 1.**
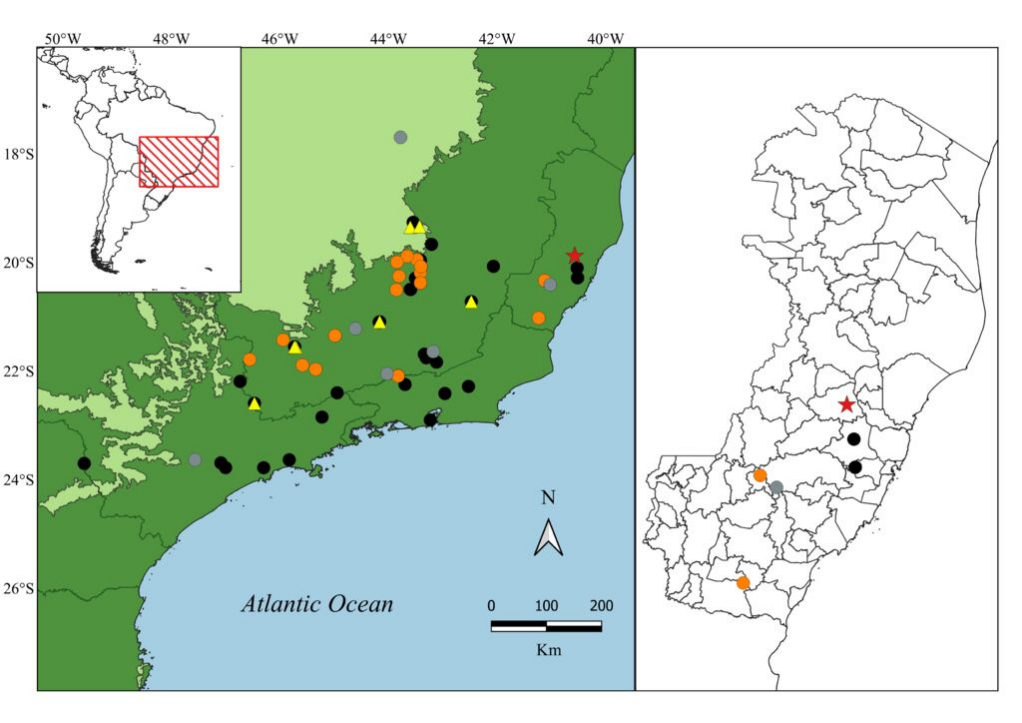
On the upper left corner - map of South America, highlighting the geographic distribution of *Heterodactylusimbricatus* in Brazil (red rectangle). In the centre - occurrence points of *H.imbricatus* in Brazil. On the right - map of State of Espírito Santo showing the cities in which *H.imbricatus* occur. Black circles = GBIF and SiBBr data; Orange circles = data from scientific literature (Tonini et al. 2010, Novelli et al. 2011, Mol et al. 2021); Grey circles = data from grey literature (Management Plans and Thesis - CEPEMAR 2004, ICMBio - MMA 2012, Stroppa 2012, CERN 2018, Lumiar Consultoria e ou Assessoria 2019,FAI UFSCar 2021); Yellow triangles = iNaturalist Research grade data; Red star = new occurrence point for the Municipality of Santa Teresa (present study). GBIF = Global Biodiversity Information Facility (www.gbif.org) and SiBBr = Sistema de Informação sobre a Biodiversidade Brasileira (www.sibbr.gov.br)

**Figure 2.**
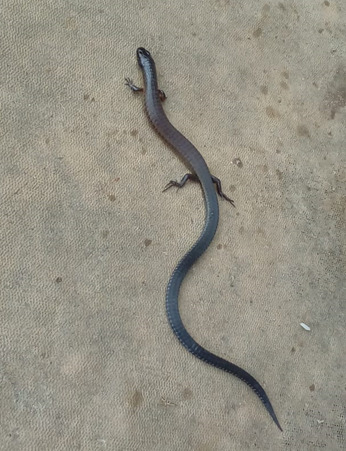
Adult of *Heterodactylusimbricatus* (dorsal view) recorded by a volunteer citizen at her residence, near the protected area “Reserva Biológica Augusto Ruschi”, Municipality of Santa Teresa, State of Espírito Santo, Brazil.

On the morning of 30 August 2019, at 10:20 am, a specimen of *H.imbricatus* was photographed with a smartphone by a local volunteer near the protected area “Reserva Biológica Augusto Ruschi”, Municipality of Santa Teresa, State of Espírito Santo, south-eastern Brazil (19°52’54.4”S, 40°34’27.7”W; 790 m alt.; Fig. 1). The adult specimen of *H.imbricatus* was observed in exposed soil, near the volunteer’s residence, located in an anthropized area, surrounded by agricultural crops, such as coffee and eucalyptus cultivations. The photograph and geolocation of the specimen (Fig. 2) was sent via WhatsApp by the volunteer to the Bromelias Project coordinator.

*Heterodactylusimbricatus* was previously recorded in five municipalities in the State of Espírito Santo: Santa Leopoldina (ZUEC-REP 1455; collected by J. L Helmer & C. Zamprogno in 1982), Venda Nova do Imigrante (MZUSP 88147; Rodrigues et al. 2009a), Cariacica (Tonini et al. 2010), Mimoso do Sul (Oliveira et al. 2020) and Domingos Martins (CEPEMAR 2004). The volunteer’s record represents a new locality for *H.imbricatus*, being the sixth for the State of Espírito Santo, extending its distribution by 25 km to the north of the State, in a new municipality (Santa Teresa), representing an advance in the understanding of the geographic distribution of the species. It is noteworthy that this is the first record of this species for the State of Espírito Santo, based on citizen science data. The Municipality of Santa Teresa is the target of several works in different fields of biology and one of the most frequently sampled areas in the State. It comprises a high diversity of taxa such as amphibians, birds, butterflies, plants and small mammals (Thomaz and Monteiro 1997, Brown and Freitas 2000, Passamani 2000, Simon 2000, Ferreira et al. 2019). Despite this, there are few studies focused on inventorying reptile species, leaving large gaps in knowledge about the geographic distribution of species.

## Data resources

The data underpinning the analysis reported in this paper are deposited at GBIF, the Global Biodiversity Information Facility, https://ipt.pensoft.net/resource?r=citizen_science_heterodactylus.

## Taxon treatments

### Heterodactylus imbricatus

Spix, 1825

B8BD5816-3CD7-52CA-B953-56F878CCC72C

#### Materials

- **Type status:** Other material. **Occurrence:** occurrenceID: 3F14EFEE-48EB-55EC-9A51-25EE0D9F01BF; **Location:** locationID: Reserva Biológica Augusto Ruschi; higherGeographyID: **TGN: 9158266**; higherGeography: South America, Brazil, Espírito Santo; continent: South America; country: Brazil; countryCode: Brazil/BRA; stateProvince: Espírito Santo; municipality: Santa Teresa; verbatimElevation: 790 m; locationAccordingTo: Getty Thesaurus of Geographic Names", "GADM"; verbatimCoordinates: 19 52 54.4S 40 34 27.7W; verbatimLatitude: 19 52 54.4S; verbatimLongitude: 40 34 27.7W; verbatimCoordinateSystem: degrees minutes seconds; verbatimSRS: unknown; georeferencedBy: Cássio Zocca (CZ); georeferenceVerificationStatus: verified by curator; **Record Level:** type: StillImage

#### Description

An adult specimen of *Heterodactylusimbricatus* (Fig. 2)

#### Taxon discussion

*Heterodactylusimbricatus* Spix, 1825 is a species of the order Squamata, of the family Gymnopthalimidae Fitzinger, 1826, inserted in the tribe Heterodactylini (Goicoechea et al. 2016).

*Heterodactylusimbricatus* is restricted to areas with a cold climate associated with high elevations and mountainous areas of south-eastern Brazil and is usually found in leaf litter (Dixo and Verdade 2006, Rodrigues et al. 2009a). Initially, the known distribution of this species was restricted to areas of the Atlantic Forest, but Novelli et al. (2011) recorded its occurrence in the domain of the Cerrado biome.

## Discussion

The record of *Heterodactylusimbricatus* by a citizen scientist shown in the present study evidences how easy it can be for those people with access to internet to explore the wildlife around them and contribute to expanding our knowledge about biodiversity. This result is in accordance with other published works, which demonstrated citizen science can contribute to a rapid accumulation of knowledge about the distribution of reptile species, including rare, endemic and/or endangered species (Price and Dorcas 2011, Theobald et al. 2015, Phillips 2016, Gómez-Hoyos et al. 2018).

*Heterodactylusimbricatus* is a very poorly-known species due to its fossorial habit. By publishing the records of volunteer citizens, we hope that more people will contribute to increase the knowledge of biodiversity in the mountainous region of Espírito Santo and expand our collective knowledge (Costello et al. 2013).

We believe it is vital for local communities near protected areas, as well as for policy-makers and managers, to comprehend the significance of these initiatives and taxonomic groups in conserving habitats, biodiversity and ecosystem services (Alves 2012). We advocate for the initiation of citizen science projects in areas neighbouring protected zones, actively involving the local communities, following the example of Projeto Bromelias.

## Supplementary Material

XML Treatment for Heterodactylus imbricatus
